# The Characterization of Two-Component System PmrA/PmrB in *Cronobacter sakazakii*

**DOI:** 10.3389/fmicb.2020.00903

**Published:** 2020-06-17

**Authors:** Jingjing Hua, Xiangyin Jia, Liang Zhang, Yanyan Li

**Affiliations:** ^1^National Engineering Laboratory for Cereal Fermentation Technology, Jiangnan University, Wuxi, China; ^2^State Key Laboratory of Food Science and Technology, Jiangnan University, Wuxi, China; ^3^Key Laboratory of Structural Biology of Zhejiang Province, School of Life Sciences, Westlake University, Hangzhou, China

**Keywords:** PmrA/PmrB two-component system, *Cronobacter sakazakii*, LPS, lipid A, polymyxin B

## Abstract

*Cronobacter sakazakii* is an opportunistic Gram-negative pathogen that could cause meningitis and necrotizing enterocolitis. Several Gram-negative bacteria use the PmrA/PmrB system to sense and adapt to environmental change by resistance to cationic antimicrobial peptides of host immune systems. The PmrA/PmrB two-component system regulates several genes to modify LPS structure in the bacterial outer membrane. The role of PmrA/PmrB of *C. sakazakii* has been studied within the current study. The results suggest that PmrA/PmrB plays a crucial role in modifying LPS structure, cationic antimicrobial peptide susceptibility, cell membrane permeability and hydrophobicity, and invading macrophage.

## Introduction

*Cronobacter sakazakii* is a Gram-negative pathogen that can be isolated from contaminated infant formula. *C. sakazakii* infection may cause bacteremia, septicemia, necrotizing enterocolitis, and other serious diseases (Chenu and Cox, 2009). Invading the intestinal cells and surviving within macrophage are the main infective characteristics with *C. sakazakii* (Forsythe et al., 2014). The outer membrane protein OmpA and efflux operon gene cluster *cusA*, *cusB*, *cusC*, and *cusR* were previously characterized as the virulence markers that associated with neonatal infections (Jaradat et al., 2014). More regulatory systems and virulence factors need to be identified in *C. sakazakii*.

Lipopolysaccharide (LPS) is the main constituent of the outer leaflet of the outer membrane of Gram-negative bacteria (Raetz and Whitfield, 2002). Lipid A is the biologically active component of LPS, which can be recognized by the innate immune system through TLR4 (Wang et al., 2010). It triggers an inflammatory response with the production of a large number of cytokines and even leads to septic shock or death (Wang B. et al., 2015). In *C. sakazakii*, the backbone of the lipid A general structure is a hexa-acylated β-1′, 6′-linked disaccharide of glucosamine, which has phosphate groups at the 1 and 4′ positions, 3-hydroxymyristate (3-OH C14:0) at the 2 and 3′ positions, and secondary fatty acid derivatives at the 2′b (C14:0) and 3′b (C12:0 or C14:0) positions (Li et al., 2016; Jia et al., 2018). Interestingly, the structure of lipid A can be modified with hydrophobic or hydrophilic groups to adapt to the change of environment. The phosphoethanolamine (pEtN) modification of lipid A was observed in *C. sakazakii* under low-pH conditions in 2016 (Liu et al., 2016). The modification contributes to the bacteria’s resistance to antimicrobial agents and, hence, to influence the ability of the host cell invasion. Currently, the lipid A modification in *C. sakazakii* has not been well characterized, and this might provide important information in terms of understanding the mechanism of bacterial infection and virulence.

Two-component regulatory systems (TCS) are quite important in regulating the virulence determinant of a lot of bacterial pathogens, which consist of sensor kinases and response regulators. The TCS PmrA/PmrB has been identified in a large amount of bacterial species, such as *Yersinia pestis* (Winfield et al., 2005), *Salmonella enterica* (Gunn, 2008), *Escherichia coli* (Hagiwara et al., 2004; Winfield and Groisman, 2004), *Klebsiella pneumonia* (Mitrophanov et al., 2008), *Citrobacter rodentium* (Viau et al., 2011), and *Pseudomonas aeruginosa* (McPhee et al., 2006). The TCS PmrA/PmrB of *Salmonella enterica* serovar Typhimurium encodes products with a sequence similarity to DNA binding response regulators and autophosphorylatable histidine kinases, respectively. It governs resistance to polymyxin B by controlling transcription of the 4-aminoarabinose biosynthetic genes (Wosten and Groisman, 1999). The TCS PmrA/PmrB of *Legionella pneumophila* triggered by acidity has a global effect on gene expression and is required for the intracellular proliferation of *Legionella pneumophila* within human macrophages and protozoa (Al-Khodor et al., 2009). The TCS PmrA/PmrB of *Escherichia coli*, also called the BasS–BasR system, is essential for iron-dependent induction of the *yfbE* operon, which is implicated in the modification of LPSs (Hagiwara et al., 2004). Briefly, PmrA/PmrB is located in the *pmrCAB* operon (Roland et al., 1993) and consists of a response regulator PmrA, a sensor kinase PmrB (Gunn, 2008; Chen and Groisman, 2013) and a pEtN transferase PmrC (Zhou et al., 2001; Lee et al., 2004; Murray et al., 2007). When bacteria lives at low pH, it is important to modify the pEtN of lipid A. The modification of pEtN increases bacteria resistance to cationic antimicrobial peptides (CAMPs) to maintain the bacterial infection in host cells. The effect of the *pmrA* gene in PmrA/PmrB on biofilm formation has been investigated (Bao et al., 2017). The *pmrA* gene may cause inhibition in biofilm formation. During biofilm formation, the *pmrA* gene may function at induction in biomass and inhibition in viability (Bao et al., 2017).

To investigate the role of PmrA/PmrB in *C. sakazakii*, we studied how pH affects *pmrA* on lipid A modification, cationic antimicrobial peptide susceptibility, cell membrane permeability and hydrophobicity, and invading macrophage. A pmrA-related mutant was generated under different pH conditions. The effect of environmental pH in lipid A modification is identified, and a *pmrA-*related mutant is generated. The *pmrA* mutant transcriptome is used to study all the gene regulations at the transcription level as compared to the wild type *C. sakazakii*.

## Materials and Methods

### Bacterial Strains and Growth Condition

The related bacterial strains and plasmids in this research are listed in Table 1. *C. sakazakii* strains and other strains were grown in Luria Bertani media (LB) (Zhang et al., 2018) at 37°C. If required, 30 μg/mL kanamycin or 100 μg/mL ampicillin was included in the medium. Strains containing the plasmid pKD46 that was temperature-sensitive were grown at 30°C, and plasmid pKD46 was cured when cells were grown at the high temperature 42°C.

**TABLE 1:** Bacterial strains and plasmids used in this study.

**Strains and plasmids**	**Description**	**Source**
*E. coli* JM109	*rec*A1 *end*A1 *gyr*A96 *thi*-1 *hsd*R17 *sup*E44 *rel*A1Δ(*lac-pro*AB)/F’ [*tra*D36 *pro*AB + lac I^q^ *lac*ZΔM15]	Labatory strain
BAA894	wild type *C. sakazakii* ATCC BAA894	Labatory strain
BAA894 pKD46	BAA894 harboring pKD46	Labatory strain
BAA894 Δ *pmrA*	BAA894 mutant with deletion of *pmrA*	This study
W3110/pWSK29-*pmrAB*	W3110 harboring pWSK29-*pmrAB*	This study
W3110/pWSK29-*pmrA*	W3110 harboring pWSK29-*pmrA*	This study
BAA894Δ*pmrA*/ pWSK29-*pmrA*	BAA894 mutan harboring pWSK29-*pmrA*	This study
BAA894/pWSK29-*pmrAB*	BAA894 harboring pWSK29-*pmrAB*	This study
Plasmids pWSK29	Low copy vector	Cai et al., 2013
pKD46	ParaBγβ exo, Repts, AmpR	Datsenko and Wanner, 2000
pKD-Cre	ParaB cre, Repts, AmpR	Datsenko and Wanner, 2000
pBlueScript II SK+	Cloning vector, ColE1, *lacZ*, AmpR	Stratagene
pDTW202	loxPLE-*kan*-loxPRE, AmpR, KanR	Datsenko and Wanner, 2000

### Construction of *C. sakazakii* BAA894/pWSK29*-pmrA/pmrB*

*pmrA* and *pmrB* (*ESA_RS16430*/*16435*) genes were amplified by PCR, taking the genome of *C. sakazakii* BAA894 (Kucerova et al., 2010) as a template. There was an *Xba*I site in the forward primer, and there was an *Xho*I site in the reverse primer. The PCR product was purified, digested with *Xba*I and *Xho*I, and ligated into the digested vector pWSK29. The pWSK29 expression vector that contains the T3/T7 lacZ operon on a replicon was induced by isopropyl-β-D-thiogalactopyranoside (IPTG). The constructed plasmid, designated pWSK29-*pmrAB*, was transformed into *C. sakazakii* BAA894, resulting in the strain *C. sakazakii*/pWSK29-*pmrA.*

### Construction of *pmrA* Knockout Mutant

To knock out *pmrA* in *C. sakazakii*, the upstream and downstream fragments of the genes were amplified by PCR. *pmrA*-U-F*/pmrA*-U-R primers were used for amplifying the upstream fragments of *pmrA*. *pmrA*-D-F*/pmrA* -D-R primers were used for obtaining the downstream fragments of *pmrA*. The kan-loxP-F and kan-loxP-R primers were used to amplify the DNA fragment loxP-kan-loxP, which contains the kanamycin resistance gene *kan* from pDTW202. The upstream PCR fragment was digested using *Pst*I and *Bam*HI, and the downstream PCR fragment was digested using *Xho*I and *Xba*I. PCR product of loxP*-kan-*loxP was digested using *Bam*HI and *Xba*I. The digested loxP*-kan-*loxP upstream and downstream fragments of *pmrA* were cloned into pBlueScript II SK, which was digested using *Pst*I and *Xho*I, constructing the plasmid pBS- *pmrA*, carrying the knockout fragment *pmrA* U-loxP-*kan*-loxP-*pmrA* D.

*pmrA* in the chromosomes of *C. sakazakii* BAA894 was removed by knockout fragment *pmrA* U-loxP-*kan*-loxP-*pmrA* D via Red recombination. First, the plasmid pKD46 was transformed into *C. sakazakii* BAA894; then, taking pBS-*pmrA* as the temple, the knockout fragment *pmrA* U-loxP-*kan*-loxP-*pmrA* D was amplified and transformed into the cells. With the expression of Red enzymes from pKD46, the DNA fragment loxP-*kan*-loxP was used to substitute the *pmrA* gene in the chromosome. Cells were cultured on LB plates with 30 μg/mL kanamycin to select the correct transformants, and the plasmid pKD46 was cured by culturing bacteria at 42°C. After which, the plasmid pKD-Cre was transformed into the bacterial cells, whose loxP recombinase Cre removed the *kan* gene previously inserted into the *C. sakazakii* chromosome. Then, the plasmid pKD-Cre was cured by growing bacteria at 42°C to obtain the *pmrA* deletion mutant strain *C. sakazakii*Δ*pmrA*. Table 1 shows the plasmids and mutants mentioned in this research.

### Isolation of Lipid A

Lipid A of *C. sakazakii* was isolated with the Bligh-Dyer method (Wang X. et al., 2015; Liu et al., 2016). Specifically, overnight culture was inoculated into 250-mL cultures, in which the initial OD_600_ was 0.02, and then cells grew to an OD_600_ of 1.0. All *C. sakazakii* cells were harvested by centrifugation at 4000 rpm for 30 min and then washed with ddH_2_O twice. The cell pellet was first suspended with 76 mL of mixture containing chloroform/H_2_O/methanol (1:0.8:2 v/v/v). The insoluble debris was then collected and washed with 60 mL mixture. The debris was heated and suspended with 12.5 mM sodium acetate (pH 4.5) by boiling water bath for 30 min. The suspension was mixed with methanol and chloroform and the mixture containing suspension/methanol/chloroform (27:30:30 v/v/v). The final mixture was centrifuged to remove its lower phase containing lipid A, which was used to extract lipid A with a rotary evaporator.

### Mass Spectrum Analysis

All the mass spectra of *C. sakazakii* lipid A samples were obtained from a Waters SYNAPT mass spectrometer, which contains an electrospray ionization (ESI) source. ESI/MS in the negative ion mode was performed to detect lipid A samples, which were dissolved in chloroform (Raetz and Whitfield, 2002). The instrument was calibrated with sodium formate. ESI/MS was performed at −80 V, and its collisional activation of ions was carried out at −8 V. MassLynx V4.1 software was used to acquire and analysis data.

### MIC Assay of Cationic Antimicrobial Peptides

The minimum inhibitory concentrations (McPhee et al., 2006) of CAMPs susceptibility were determined by a twofold serial dilution method of polymyxin B (from 1000 to 0.095 μg/mL) and polymyxin E (colistin) (from 1000 to 0.45 μg/mL). Overnight culture was inoculated in a 96-well plate and grown at 37°C. The media for dilutions of polymyxin B and E was the used LB broth. By adding 100 μL per well in the 96-well plate, the overnight bacteria culture suspension was diluted 500 times with OD_600_ = 0.5. If the culture was significantly cloudy, the growth was rated positive. For two different occasions, each test was performed three times (Wang Z. et al., 2015).

### Membrane Permeability Assay

The outer membrane permeability (Wang et al., 2014) was detected by fluorescent probe 1-N-phenylnaphthylamine (NPN) access assay (Helander and Mattila-Sandholm, 2000). Briefly, *C. sakazakii* BAA894 was grown overnight in LB medium, and 1.5 ml cells were first harvested by centrifugation at 12,000 rpm for 3 min, then washed twice with potassium phosphate buffer (PBS, 50 mM, pH 7.4). The PBS buffer was used to adjust the OD_600_ to 0.5. A fluorescence spectrophotometer (650–660, Hitachi, Japan) whose widths, excitation, and emission wavelengths were set as 5, 350, and 420 nm, respectively, was used to monitor the fluorescence of the mixture of 1.92 mL of cell suspension (OD_600_ = 0.5) and 80 μL NPN (1 mM) (Wang et al., 2014).

### Surface Hydrophobicity Assay

The surface hydrophobicity of cells was determined according to a surface hydrophobicity assay involving the method of Zavaglia et al. (2002) and (Wang et al., 2014). Briefly, the bacteria were collected from overnight culture and resuspended with PBS. After being washed twice by PBS (pH 7.4), the OD_600_ of the culture was adjusted to 0.5. The mixture of 2 mL bacterial suspension and 800 μL xylene was incubated at room temperature for 3 h, which obtained the aqueous phase. The OD_600_ of the aqueous phase was recorded as *A*, and the value of [(0.5 - *A*)/0.5] × 100 represents the surface hydrophobicity of the bacterial.

### Macrophages Infection

RAW264.7 macrophage cells were seeded in 96-well plates and were grown in Phenol red-free DMEM with 10% fetal bovine serum at 37°C with 5% CO_2_. The bacteria was grown in LB (pH 5.0) broth and added into the wells to adjust the macrophage:bacteria ratio of 1:100. After infection for 2 h, culture supernatant was removed and cells were washed with warm PBS four times to remove extracellular bacteria. Afterward, fresh, antibiotic-free media was added to wells. At a desired time point, intracellular bacteria were harvested by adding an equal volume of 0.5% Triton X-100 into wells and left at 37°C and 5% CO_2_ for 5 min. To detach cells from wells, a pipette was used to scrape out cells. Bacterial number was determined by serial dilution plating onto LB plates.

### RNA Sequence

The RNA sequence of cells was conducted according to the manufacturer’s protocol. Briefly, the cells were collected from the logarithmic phase at pH 5.0; DNA and rRNA were then removed from the total RNA with a kit (Dongsheng Biotech, Guangzhou, China). First, the mRNA was broken into short fragments; then cDNA was synthesized by taking the disrupted mRNA as template. Double-stranded cDNA was synthesized by a two-strand synthesis reaction system. The double-stranded cDNA was purified with QiaQuick PCR kit (QIAGEN, Hamburg, Germany). Next, cohesive ends were repaired. The base “A” was added to the 3′ end of the cDNA, and products were ligated to the sequencing adapter; then suitable fragments were selected by agarose electrophoresis, and finally, PCR amplification was performed. In the quality control steps, an ABI StepOnePlus Real-Time PCR System and an Agilent 2100 Bioanaylzer were used for quantification and qualification of the sample library, which was prepared to be sequenced by Illumina HiSeqTM 2000.

### RNA Extraction and Transcriptional Analysis Through RT-PCR

Ten DEGs relevant to phenotype were selected to verify the RNA-seq data by the quantitative mRNA transcripts with real-time polymerase chain reaction (qRT-PCR) using the ABI Step One RT-PCR System (Applied Biosystems, CA) according to the manufacturer’s instructions. Total RNA samples were extracted using an RNA extraction kit (BioFlux, China). Then RNase-free DNase I was used to remove DNA contamination. Transcription of 500 ng RNA into cDNA was performed using a Revert Aid^TM^ First Strand cDNA synthesis kit (Fermentas, Shanghai, China) with random hexamer primers. Primers for detection of various genes are listed in Table 4. 16S rRNA gene was used as an internal control for quantification of relative gene expression. Each qRT-PCR reaction was conducted in a final volume of 50 μL. The thermal cycling profile was as follows: 94°C for 1 min, followed by 40 cycles of 94°C for 10 s, 55°C for 30 s, and 68°C for 15 s. Sterile water was used as negative control samples. The cycle threshold values (CT) were determined, and the 2^–ΔΔCT^ method (Livak and Schmittgen, 2001) with *16S rRNA* as the reference gene was used to calculate the relative fold differences. This experiment was repeated three times.

## Results and Discussion

### Identification of the Genes Encoding PmrA/PmrB in *C. sakazakii*

The PmrA/PmrB two-component system involved in *S. typhimurium* was activated directly by the existing iron in the culture medium and indirectly through the PhoP/PhoQ and PmrA/PmrB signal systems at a low magnesium concentration or low pH (Wosten et al., 2000; Gibbons et al., 2005). Phosphoethanolamine (pEtN) is incorporated to lipid A when bacteria are grown under low pH conditions in *S. typhimurium*. The pEtN modification of BAA894 lipid A can increase the resistance to CAMPs and reduce recognition and lethality by the host innate immune system (Liu et al., 2016). To identify *C. sakazakii* genes that are responsible for the PmrA/PmrB regulatory system, the amino acid sequence of the *S. typhimurium* PmrA, PmrB, PmrC protein was used as the query to perform a BLASTp search of the BAA894 genome.

The *C. sakazakii* BAA894 *ESA_RS16430* showed 56.31% identity to *pmrA* in *S. typhimurium*, and *ESA_RS16435* exhibited 46.43% identity with *pmrB* in *S. typhimurium*. In the genomes of *S. typhimurium*, *eptA* (*pmrC*) locates in the same operon with *pmrA* and *pmrB* (Lee et al., 2004), and the gene expression is controlled by PmrA (Wosten and Groisman, 1999). In the genome of *C. sakazakii* BAA894 (Joseph et al., 2012), *ESA_RS16425* under the same operon with *pmrAB* showed 82.14% identity to *dacB* from *S. typhimurium.* Another gene of *ESA_ RS09200*, which is outside of the operon, shows identity to *pmrC* in *C. sakazakii.* The functional relationship between *ESA_RS16425* and *pmrA/pmrB* is unknown. The gene organization of *C. sakazakii* is different with the *pmrCAB* operon identified in *S. typhimurium* and *E. coli*. The *pmrAB* overexpression and *pmrA* deletion strains were generated to study the function and regulation mechanism.

### The Effect of PmrA/PmrB on Lipid A Structure Modification in *C. sakazakii*

**FIGURE 1:**
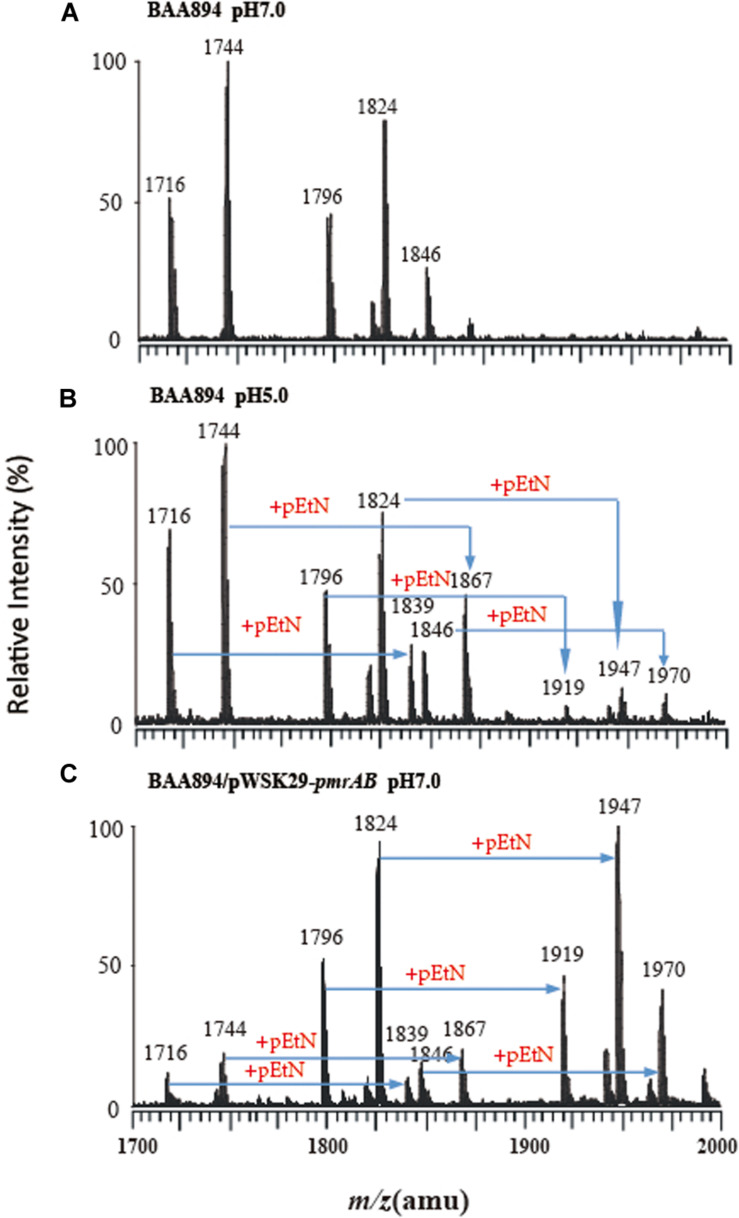
Mass spectrometry analysis of lipid A isolated from *C. sakazakii* and *C. sakazakii/*pWSK29-*pmrAB*. **(A)** *C. sakazakii* grown at pH 7.0. **(B)** *C. sakazakii* grown at pH 5.0. **(C)** *C. sakazakii*/pWSK29-*pmrAB* grown at pH 7.0.

To investigate the PmrA/PmrB effect on the lipid A structure in *C. sakazakii*, *pmrAB* were co-overexpressed and the *pmrA* mutant was generated in *C. sakazakii*. The lipid A was extracted from wild-type *C. sakazakii* BAA894 at pH 7.0 and pH 5.0, respectively (Figures 1A,B), and *pmrAB* overexpression stain *C. sakazakii*/pWSK29-*pmrAB* (Figure 1C) at pH 7.0 by the Bligh-Dyer method (Gunn et al., 2000; Bengoechea et al., 2003; Townsend et al., 2007; Wang X. et al., 2015). ESI/MS was used to analyze the extracted lipid A.

The lipid A from *C. sakazakii* BAA894 at pH 7.0 showed five major peaks at *m/z* 1716, 1744, 1796, 1824, and 1846 in the spectrum. The ions at *m/z* 1796 and 1824 are the [M–H]^–^ ions of the two lipid A molecules, which are the backbone of the lipid A general structure (Li et al., 2016). The minor peaks at *m/z* 1716 and 1744 are the monophosphorylated form of lipid A (Helander and Mattila-Sandholm, 2000). The peak at *m/z* 1846 was generated from the sodium adduct of the ion at *m/z* 1824 (Figure 1A). The lipid A ions at pH 5.0 showed five additional peaks at *m/z* 1839, 1867, 1919, 1947, and 1970 (Figure 1B). All these ions with 123 amu higher *m/z* suggested the addition of a pEtN group into lipid A. The low pH condition upregulated the *pmrC* gene expression.

The lipid A isolated from *pmrAB* overexpression strain *C. sakazakii*/pWSK29-*pmrAB* at pH 7.0 also showed five additional peaks at *m/z* 1839, 1867, 1919, 1947, and 1970 with higher 123 amu compared to the wild-type grown at pH 7.0. In addition, the relative intensity of these peaks was also much higher than that of the wild-type grown at pH 5.0 (Figure 1C). The results suggest that the overexpression of *pmrAB* boosted the pEtN modification in lipid A. The overexpression of *pmrAB* likely upregulated the *pmrC* gene transcription level and, hence, resulted in the modification of lipid A with the addition of the pEtN group. Compared to the wild-type grown at pH 7.0, the *pmrA* mutant did not show a significant difference in the lipid A profile, and there was no lipid A modification found as well.

### PmrA/PmrB-Mediated Lipid A Modification Increases the Resistance to Cationic Antimicrobial Peptides

The PmrA/PmrB is the major regulator of LPS-modified genes in *S. typhimurium* and *E. coli*. The phosphate groups on the surface of the bacteria would be neutralized by the modification of pEtN on lipid A and, hence, become negatively charged and could interact with CAMPs, such as Polymyxin B and colistin (Anandan et al., 2016). To study whether *pmrAB*-mediated pEtN modification of lipid A in *C. sakazakii* BAA894 alters the bacterial resistance to CAMPs (Liu et al., 2016), MIC of *C. sakazakii* wild-type strain, *pmrA* mutant strain, and *pmrAB* overexpressed strain grown at pH 7.0 and 5.0 to polymyxins B and colistin were investigated (Table 2). *C. sakazakii* BAA894 cells grown at pH 5.0 were highly resistant to CAMPs compared to the cells grown at pH 7.0; the MIC of polymyxin B increased from 0.39 to 17.5 μg/mL, and the MIC of polymyxin E increased from 0.45 to 35 μg/mL. When grown at pH 5.0, the MIC of polymyxin B and colistin of the *pmrA* mutant strain were 6.5 μg/mL and 4 μg/mL, respectively. The result was still higher than that of the wild-type strain (Table 2). Compared to the BAA894 grown at pH 7.0, the MIC of *C. sakazakii* BAA894/pWSK29-*pmrAB* to polymyxin B and colistin grown at pH 5.0 were higher, which were increased to 280 and 560 μg/mL, respectively. Complementation of BAA894Δ*pmrA* and pWSK29-*pmrA*, its MIC to polymyxin B and colistin returned to wild-type levels at pH 7.0 or 5.0. According to our study, pEtN modification of lipid A at acidic pH can improve bacteria resistance ability to CAMPs. On the minimal inhibitory concentrations table (Table 2), we found that *C. sakazakii* BAA894/pWSK29-*pmrAB* showed the highest resistance ability to CAMPs at pH 5.0, but remained at the same resistance level to CAMPs when compared with BAA894 at pH 7.0. The results suggested that the PmrA were related to the resistance to CAMPs, and PmrA/PmrB also influenced the resistance to CAMPs at acidic pH.

**TABLE 2:** The minimal inhibitory concentrations of mutants derived from BAA894 cultured in pH 7.0 and pH 5.0.

**Strain**	**pH**	**Polymyxin B MIC** **(μg/mL)**	**Polymyxin E MIC** **(μg/mL)**
*C. sakazakii*	pH 7.0	0.390.1	0.450.1
	pH 5.0	17.52.5	352.5
*C. sakazakii*Δ*pmrA*	pH 7.0	0.390.1	0.450.1
	pH 5.0	6.51.0	4.01.0
BAA894/pWSK29-*pmrAB*	pH7.0	0.390.1	0.450.1
	pH5.0	28020	56035
*C. sakazakii*Δ*pmrA*	pH7.0	0.390.1	0.450.1
complemented	pH5.0	17.52.5	352.5

*The values represent the mean ± SD of results from three independent experiments.*

### PmrA/PmrB-Mediated Lipid A Modification Changes the Cell Surface Properties of *C. sakazakii*

**FIGURE 2:**
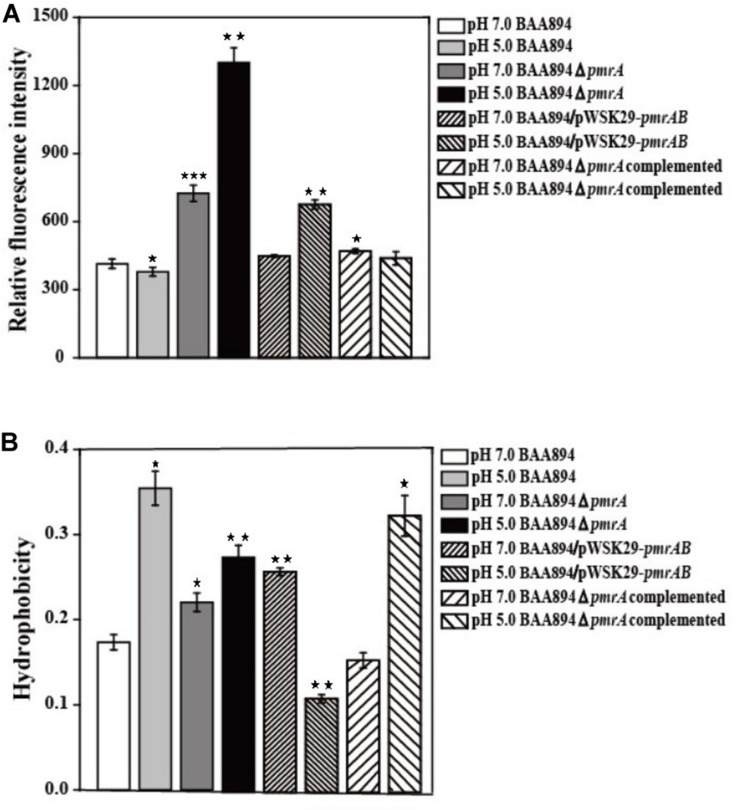
The cell surface properties of mutants derived from BAA894 cultured in pH 7.0 and pH 5.0. **(A)** Membrane permeability assay. **(B)** Hydrophobicity assay. The values represent the mean ± SD of results from three independent experiments. The data were analyzed by one-way analysis of variance. Statistically significant differences between the mutants in pH 7.0 and pH 5.0 and wild strains BAA894 in pH 7.0: *p < 0.05; **p < 0.01; and ***p < 0.001.

It has been shown previously that the OM permeability of Gram-negative bacteria is correlated with the structure of LPS (Bengoechea et al., 2003). Lipid A is the important hydrophobic component of LPS, which is the major component of the outer membrane; the structure changes of the lipid A might influence the cell membrane properties (Raetz and Whitfield, 2002; Bengoechea et al., 2003). Since our research suggested that the overexpression of *pmrAB* boosted the pEtN modification in lipid A, the OM permeability and hydrophobicity might be influenced through the change of structure of the LPS and outer menmrane. The characteristics of the outer membrane, including the permeability and hydrophobicity of mutants cells (Lehner et al., 2005) were evaluated (Figure 2).

When compared to the *C. sakakzakii* BAA894 wild-type strain cultured at pH 7.0, the membrane permeability of BAA894 cultured at pH 5.0 slightly decreased. In comparison with the BAA894 wild-type strain, the membrane permeability of *pmrA* mutant strains cultured at pH 7.0 and pH 5.0 increased by twofold and threefold, respectively (Figure 2A). Compared with BAA894 grown at pH 5.0, the membrane permeability of BAA894/pWSK29-*pmrAB* cultured at pH 7.0 was slightly increased while the one that was cultured at pH 5.0 increased by 1.8-fold. Under the pH 7.0 or pH 5.0 condition, the *pmrA* overexpressed strain restored the membrane permeability to the wild-type levels. The PmrA/PmrB system-mediated lipid A modification changed the structure of the LPS and reduced outer membrane permeability. These results suggest that the *pmrA* and PmrA/PmrB system have an influence on membrane permeability.

The cell surface hydrophobicity of the *pmrA* mutant strain was essentially the same at pH 7.0 and pH 5.0. However, the cell surface hydrophobicity of the BAA894 wild-type strain and BAA894Δ*pmrA* complementation strain under pH 5.0 was twofold higher than at pH 7.0 (Figure 2B). Meanwhile, compared with the BAA894 wild-type strain, the cell surface hydrophobicity of BAA894/pWSK29-*pmrAB* cultured at pH 7.0 and pH 5.0 increased by 1.5- and 1.6-fold. These results suggest that modification of pEtN in lipid A might change the characteristics of the outer membrane by increasing the surface hydrophobicity of cells.

### PmrA/PmrB Inhibits the Ability to Invade and Survive in Macrophage

**FIGURE 3:**
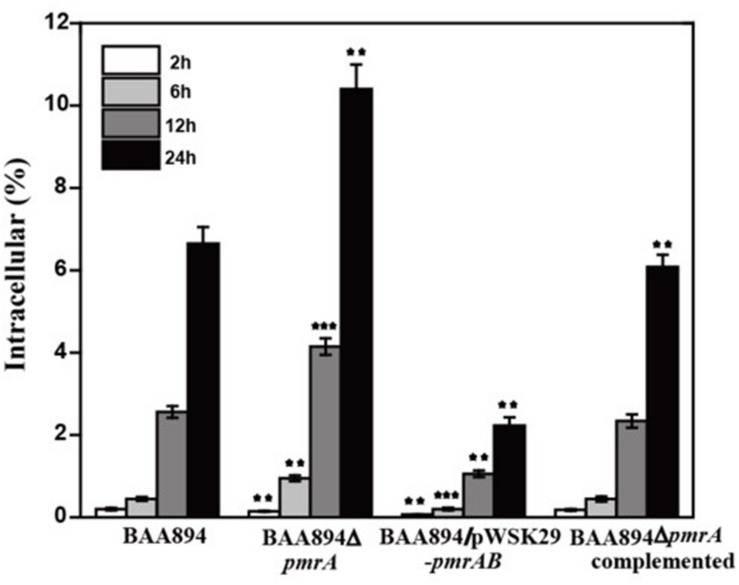
Survival and replication in RAW264.7 macrophages. Results are presented as the percentage of the initial inoculum that was intracellular. The values represent the mean ± SD of results from three independent experiments. The data were analyzed by one-way analysis of variance. Statistically significant differences between the mutants and *C. sakazakii* wild strain: **p < 0.01 and ***p < 0.001.

To invade microorganisms, macrophages utilize antimicrobial defense mechanisms (i.e., nutrient deprivation and oxidative burst) to eliminate harmful pathogens. The ability of bacteria to survive and replicate in immune cells provides them protection from the host immune response (Townsend et al., 2007). Some *C. sakazakii* can avoid the host immune response by exploiting immature dendritic cells and then persisting within human macrophages (Liu et al., 2016). The *pmrA* mutant strain grew faster than the BAA894 wild-type strain at pH 5.0, and more mutants were recovered from macrophage after infection for 6, 12, and 24 h. The intracellular numbers of the wild-type strain increased 2.56-fold and 6.65-fold over T12 and T24, respectively, while those of the mutant strains were 4.15-fold and 10.4-fold over T12 and T24, respectively (Figure 3). However, the growth state of *C. sakazakii* BAA894/pWSK29-*pmrAB* in macrophage was worse than that of the BAA894 wild-type strain at pH 5.0. In addition, compared with BAA894 grown at pH 5.0, the intracellular numbers of the mutant decreased to 41% and 34% over T12 and T24, respectively (Figure 3). These results demonstrate that the *pmrA* mutant increased cell invasion and replication ability, which was restored to wild-type levels by complementation. All these suggested that the PmrA/PmrB two-component system in *C. sakazakii* BAA894 was important in regulating the system for host cell invasion and replication.

### RNA-Sequencing of *C. sakazakii* pmrA Mutant

In our study, PmrA/PmrB shows influences on CAMPs, cell membrane permeability and hydrophobicity, and macrophage invasion. To verify the results, further analysis by RNA-sequencing were conducted (Table 3).

**TABLE 3:** List of the genes related to phenotype between BAA894 and pmrA mutant in pH 5.0.

**gene ID**	**5.0WT RPKM**	**5.0*pmrA* RPKM**	**Fold change**	***P* value**	**Level**
*pmrA*	90.13	0.00	0.00	*P* < 0.01	Down
*pmrB*	107.56	47.24	0.44	*P* < 0.01	Down
*phoP*	286.85	313.69	1.09	*P* < 0.01	Up
*phoQ*	175.54	134.05	0.76	*P* < 0.01	Down
*eptA*	158.65	184.59	1.16	*P* < 0.01	Up
*eptB*	425.35	334.12	0.79	*P* < 0.01	Down
*pagP*	145.53	132.29	0.91	*P* < 0.01	Down
*lpxA*	458.78	449.40	0.98	*P* < 0.01	Down
*cpxA*	224.27	141.26	0.63	*P* < 0.01	Down
*cpxR*	634.07	536.28	0.85	*P* < 0.01	Down
*marA*	29.74	22.07	0.74	*P* < 0.01	Down
*acrA*	382.12	377.03	0.99	*P* < 0.01	Down
*acrB*	228.82	210.03	0.92	*P* < 0.01	Down
*tolC*	315.09	316.41	1.00	*P* < 0.01	Up
*ramA*	87.80	74.79	0.85	*P* < 0.01	Down
*ompF*	7971.55	8755.51	1.10	*P* < 0.01	Up
*phoE*	58.84	80.38	1.37	*P* < 0.01	Up
*ompC*	11281.95	17012.45	1.51	*P* < 0.01	Up
*ESA_RS01970*	743.76	818.06	1.10	*P* < 0.01	Up
*ESA_RS03645*	61.54	131.54	2.14	*P* < 0.01	Up
*ESA_RS08640*	172.73	314.67	1.82	*P* < 0.01	Up
*ESA_RS19800*	50.24	138.89	2.77	*P* < 0.01	Up
*ESA_RS19805*	18.28	52.33	2.86	*P* < 0.01	Up
*ESA_RS11615*	23194.65	26474.44	1.14	*P* < 0.01	Up
*ompX*	3.08	3.63	1.18	*P* < 0.01	Up
*ESA_RS16175*	1.18	1.39	1.18	*P* < 0.01	Up
*ESA_RS17575*	12.88	14.19	1.10	*P* < 0.01	Up
*ESA_RS18760*	40.25	45.60	1.13	*P* < 0.01	Up
*ESA_RS18775*	27.72	35.70	1.29	*P* < 0.01	Up
*fliR*	69.80	100.78	1.44	*P* < 0.01	Up
*ESA_RS05890*	110.76	351.83	3.18	*P* < 0.01	Up
*fliS*	90.79	244.40	2.69	*P* < 0.01	Up
*fliJ*	39.00	21.21	0.54	*P* < 0.01	Down
*ESA_RS05905*	649.94	307.50	0.47	*P* < 0.01	Down

**TABLE 4:** Primers used in RT-PCR.

**Primers**	**Nucleotide sequence (5′ → 3′)**
RT-*16SrDNA*- F	CCTTACGACCAGGGCTAC
RT-*16SrDNA*-R	GACTACGACGCACTTTATGAG
RT-*pmrB*- F	GCTCGGCGGATAATCTT
RT-*pmrB*- R	GATGTGGCTTCGTCGTGAG
RT-*phoP*- F	CTGGTCGTCGAGGATAACG
RT-*phoP*- R	TGTGAAACGGCTTGGTG
RT-*phoO*- F	GAGCGTCTGGAACTGGTTTAT
RT-*phoO*- R	GGTGAGCGTTGTGCGGT
RT-*eptA*- F	AACAGAACTATTCGCTTGGC
RT-*eptA*- R	GCAGGAGCCCTCTTTACAC
RT-*pagP*- F	ACCAGCGGAATCGGGAT
RT-*pagP*- R	GCCTGGGCTTCTGTGCTT
RT-*eptB*- F	GATACGGCGACCAAACTCT
RT-*eptB*- R	TCAACTCCTGACGGCTAC
RT-*ESA-RS01970*- F	GAGCGACGAAGACAAACG
RT-*ESA-RS01970*- R	AGAGTGCCACAGGACCAC
RT-*ESA-RS19805*- F	ACCGTCTGTTGCAGGTC
RT-*ESA-RS19805*- R	TGCCGCCACATCAATACC
RT-*fliS*- F	TCAGGGTTTCAACTTCATTG
RT-*fliS*- R	CCTGGAAAGTGCGGTAAT
RT-*fliJ*- F	CGCCTGATCGACTTTGGT
RT-*fliJ*- R	ACGCTTAAAGATATGGCTG

The gene expression levels related to phenotype were between the BAA894 and *pmrA* mutant at pH 5.0. RPKM is short for reads per kilobase transcriptome per million mapped reads; it is used to calculate the expression level of the gene. Fold change represents the ratio of *pmrA* mutant to wild-type gene RPKM; *p* value represents the significance level of the hypothesis test.

**FIGURE 4:**
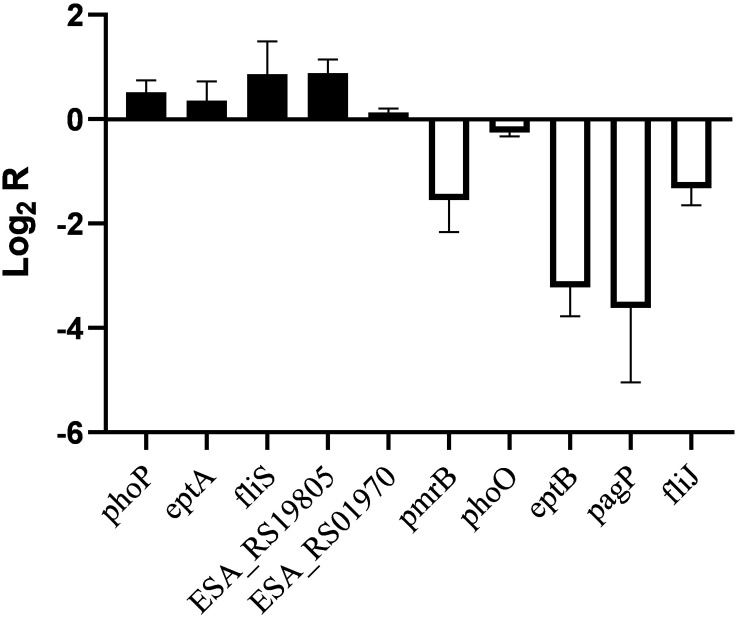
RT-PCR analysis for transcriptional levels of genes related to phenotype in *C. sakazakii* pmrA in pH 5.0, using *C. sakazakii* wild strain in pH 5.0 as the control.

Similar transcriptional levels of some key genes relevant to phenotype were also observed by using RT-PCR analysis (Figure 4). This suggests that the transcriptomic analysis used in this study is reliable.

In *Salmonella enterica*, PmrB can sense environmental stimuli directly and can initiate a cascade to phosphorylate and activate PmrA. LPS modifications mediated by PmrA/PmrB and PhoP/PhoQ systems aid in survival in host cells and in the environment (Gunn, 2008). In the *pmrA* mutant, genes (*pmrB*, *phoQ*, *pagP*, and *eptB*) of PmrA/PmrB and PhoP/PhoQ were downregulated, which indicated that the *pmrA* mutant without a complete PmrAB regulon might weaken survival in host and non-host environments. In *C. sakazakii* and *E. coli*, PmrA/PmrB and PhoP/PhoQ changed the resistance to CAMPs by modifying fatty acid chains and phosphoric acid groups of lipid A (Townsend et al., 2007; Wang X. et al., 2015; Liu et al., 2016). In the *pmrA* mutant, some of the PmrAB-regulated genes were downregulated while *eptA* and *phoP* were upregulated. Besides, genes (*lpxA*, *cpxA*, *cpxR*, *marA*, *acrA*, and *acrB*) involved in CAMPs resistance were downregulated in the *pmrA* mutant while *tolC* was upregulated. Most of the genes involved in CAMPs resistance were downregulated, which indicated that the *pmrA* mutant was weakly resistant to CAMPs.

Genes (*marA*, *ramA*, *ompF*, *phoE*, and *ompC*) are involved in regulating membrane permeability by changing the production of porin and the expression of bacterial envelope efflux pump systems (Bishop, 2005; Anne et al., 2008). In transcriptome data, genes (*ompF*, *phoE*, and *ompC*) were upregulated in *pmrA* mutant while *marA* and *ramA* were downregulated, suggesting the variation of membrane permeability.

Due to the difference in modification of LPS, cell hydrophobicity changed (Kim et al., 2010). Also, genes associated with hydrophobicity, such as *eptB*, *pagP*, were downregulated in *pmrA* mutant while *eptA* was upregulated, suggesting the variation of hydrophobicity.

Other influence factors for macrophage invasion include both virulence factors and bacterial motility (Delcour, 2009; Kim et al., 2010). Genes (*ESA_RS01970*, *ESA_RS03645*, *ESA_RS08640*, *ESA_RS19800*, and *ESA_RS1980*) involved in toxins were upregulated in *pmrA* mutant. Genes (*ompX*) involved in bacterial motility can promote bacterial adhesion and attachment to the host (Delcour, 2009). Fimbriae proteins (*ESA_RS12900*, *ESA_RS16175*, *ESA_RS17575*, *ESA_RS18760*, and *ESA_RS18775*) and flagellum proteins (*fliR*, *ESA_RS05890*, and *fliS*, except for *fliJ* and *ESA_RS059i05*) were upregulated in the *C. sakazakii pmrA* mutant. Because of the impact of virulence and bacterial adhesion, the *pmrA* mutant increased the ability of cells to invade.

According to KEGG pathway enrichment analysis, “lysine biosynthesis,” “metabolic pathways,” “biosynthesis of secondary metabolites,” and “microbial metabolism in diverse environments” were significantly enriched. In the *pmrA* mutant, most differentially expressed genes (DEGs) in “microbial metabolism in diverse environments” were downregulated. DEGs (*ESA_RS15995*, *ESA_RS17800*, and *ESA_RS18990*) involved in the “pentose phosphate cycle” were also downregulated, which indicated that the *pmrA* mutant without a complete PmrAB regulon might weaken survival in diverse environments.

## Conclusion

The PmrA/PmrB-regulated genes were identified and characterized in *C. sakazakii*. The pEtN is modified to lipid A in *C. sakazakii* when grown at a weak acid condition. The enzyme encoded by *C. sakazakii eptA* could transfer pEtN to lipid A and was affected by acidic condition. More studies indicate that the *eptA* is not completely but partially regulated by PmrA/PmrB in *C. sakakzakii* BAA894, and the PmrA/PmrB two-component system in *C. sakazakii* were involved in the lipid A structure modification.

Non-polar deletion was constructed in the *pmrAB* gene of the PmrA/PmrB, and the effect of this mutation on CAMPs resistance were measured. Negative-ion mass spectra revealed the presence of pEtN in lipid A isolated from the *pmrAB* overexpressed strain under the pH 7.0 condition. Meanwhile, we also tested mutant and wild-type strains for susceptibility to CAMPs and demonstrated the PmrA and PmrA/PmrB were related to the resistance of CAMPs. A defect of the *pmrA* gene and co-overexpression of *pmrAB* in *C. sakazakii* increased the outer membrane permeability and hydrophobicity at pH 5.0, which might improve the transmembrane capacity of antibiotics and then affect the outer membrane permeability and hydrophobicity.

*Cronobacter sakazakii* can utilize immature dendritic cells and remain in human macrophages, which indicates that *C. sakazakii* has certain immune evasion properties and can protect itself from host immune response and then reach and even penetrate the blood–brain barrier (Townsend et al., 2008; Emami et al., 2011; Wang X. et al., 2015). The BAA894 wild-type strain showed lower invasion ability than the *pmrA* mutant strain and higher invasion ability than BAA894/pWSK29-*pmrAB* under the pH 5.0 condition, suggesting that the PmrA/PmrB system is important in toxicity control in *C. sakazakii*. When *pmrA* was deleted, the bacteria showed a trend of increased toxicity and provided a survival advantage to bacteria in the host.

## Data Availability Statement

The dataset can be found in NCBI under the accession number GSE147019.

## Author Contributions

All authors listed have made a substantial, direct and intellectual contribution to the work, and approved it for publication.

## Conflict of Interest

The authors declare that the research was conducted in the absence of any commercial or financial relationships that could be construed as a potential conflict of interest.

## References

[B1] Al-Khodor S. Kalachikov S. Morozova I. Price C. T. Abu Kwaik Y. (2009). The PmrA/PmrB two-component system of *Legionella pneumophila* is a global regulator required for intracellular replication within macrophages and protozoa. *Infect Immun.* 77 374–386. 10.1128/IAI.01081-08 18936184 PMC2612241

[B2] Anandan A. Evans G. L. Condic-Jurkic K. O’Mara M. L. John C. M. Phillips N. J. (2016). Structure of a lipid A phosphoethanolamine transferase suggests how conformational changes govern substrate binding. *PNAS* 114 2218–2223. 10.1073/pnas.1612927114 28193899 PMC5338521

[B3] Anne D.-R. Bolla J. M. James C. E. Lavigne J. P. Chevalier J. Garnotel E. (2008). Membrane permeability and regulation of drug. *Curr. Drug Targets* 9 750–759. 10.2174/138945008785747824 18781921

[B4] Bao X. Jia X. Chen L. Peters B. M. Lin C.-W. Chen D. (2017). Effect of polymyxin resistance (pmr) on biofilm formation of *Cronobacter sakazakii*. *Microb. Pathog.* 106 16–19. 10.1016/j.micpath.2016.12.012 28012985

[B5] Bengoechea J. A. Brandenburg K. Arraiza M. D. Seydel U. Skurnik M. Moriyón I. (2003). Pathogenic *Yersinia enterocolitica* strains increase the outer membrane permeability in response to environmental stimuli by modulating lipopolysaccharide fluidity and lipid A structure. *Infect Immun.* 71 2014–2021. 10.1128/iai.71.4.2014-2021.2003 12654821 PMC152087

[B6] Bishop R. E. (2005). Fundamentals of endotoxin structure and function. *Contrib. Microbiol.* 12 1–27. 10.1159/000081687 15496774

[B7] Cai L. Li Y. Tao G. Guo W. Zhang C. Wang X. (2013). Identification of three genes encoding for the late acyltransferases of lipid A in *Cronobacter sakazakii*. *Mar. Drugs* 11, 377–386. 10.3390/md11020377 23434833 PMC3640386

[B8] Chen H. D. Groisman E. A. (2013). The biology of the PmrA/PmrB two-component system: the major regulator of lipopolysaccharide modifications. *Annu. Rev. Microbiol.* 67 83–112. 10.1146/annurev-micro-092412-155751 23799815 PMC8381567

[B9] Chenu J. W. Cox J. M. (2009). Cronobacter (‘*Enterobacter sakazakii*’): current status and future prospects. *Lett. Appl. Microbiol.* 49 153–159. 10.1111/j.1472-765X.2009.02651.x 19486285

[B10] Datsenko K. A. Wanner B. L. (2000). One-step inactivation of chromosomal genes in *Escherichia coli* K-12 using PCR products. *Proc. Nat. Acad. Sci. USA* 97, 6640–6645. 10.1073/pnas.120163297 10829079 PMC18686

[B11] Delcour A. H. (2009). Outer membrane permeability and antibiotic resistance. *Biochim. Biophys. Acta* 1794 808–816. 10.1016/j.bbapap.2008.11.005 19100346 PMC2696358

[B12] Emami C. N. Mittal R. Wang L. Ford H. R. Prasadarao N. V. (2011). Recruitment of dendritic cells is responsible for intestinal epithelial damage in the pathogenesis of necrotizing enterocolitis by *Cronobacter sakazakii*. *J. Immunol.* 186 7067–7079. 10.4049/jimmunol.1100108 21551359

[B13] Forsythe S. J. Dickins B. Jolley K. A. (2014). Cronobacter, the emergent bacterial pathogen *Enterobacter sakazakii* comes of age; MLST and whole genome sequence analysis. *BMC Genomics* 15:1121. 10.1186/1471-2164-15-1121 25515150 PMC4377842

[B14] Gibbons H. S. Kalb S. R. Cotter R. J. Raetz C. R. (2005). Role of Mg2+ and pH in the modification of *Salmonella* lipid A after endocytosis by macrophage tumour cells. *Mol. Microbiol.* 55 425–440. 10.1111/j.1365-2958.2004.04409.x 15659161

[B15] Gunn J. S. (2008). The *Salmonella* PmrAB regulon: lipopolysaccharide modifications, antimicrobial peptide resistance and more. *Trends Microbiol.* 16 284–290. 10.1016/j.tim.2008.03.007 18467098

[B16] Gunn J. S. Ryan S. S. Van Velkinburgh J. C. Ernst R. K. Miller S. I. (2000). Genetic and functional analysis of a PmrA-PmrB-regulated locus necessary for lipopolysaccharide modification, antimicrobial peptide resistance, and oral virulence of *Salmonella enterica* serovar typhimurium. *Infect Immun.* 68 6139–6146. 10.1128/iai.68.11.6139-6146.2000 11035717 PMC97691

[B17] Hagiwara D. Yamashino T. Mizuno T. (2004). A Genome-wide view of the *Escherichia coli* BasS-BasR two-component system implicated in iron-responses. *Biosci. Biotechnol. Biochem.* 68 1758–1767. 10.1271/bbb.68.1758 15322361

[B18] Helander I. M. Mattila-Sandholm T. (2000). Fluorometric assessment of gram-negative bacterial permeabilization. *J. Appl. Microbiol.* 88:6. 10.1046/j.1365-2672.2000.00971.x 10735988

[B19] Jaradat Z. W. Al Mousa W. Elbetieha A. Al Nabulsi A. Tall B. D. (2014). *Cronobacter* spp. - opportunistic food-borne pathogens. A review of their virulence and environmental-adaptive traits. *J. Med. Microbiol.* 63 1023–1037. 10.1099/jmm.0.073742-0 24878566

[B20] Jia X. Hua J. Liu L. Xu Z. Li Y. (2018). Phenotypic characterization of pathogenic *Cronobacter* spp. strains. *Microb. Pathog.* 121 232–237. 10.1016/j.micpath.2018.05.033 29800699

[B21] Joseph S. Desai P. Ji Y. Cummings C. A. Shih R. Degoricija L. (2012). Comparative analysis of genome sequences covering the seven cronobacter species. *PLoS One* 7:e49455. 10.1371/journal.pone.0049455 23166675 PMC3500316

[B22] Kim K. Kim K. P. Choi J. Lim J. A. Lee J. Hwang S. (2010). Outer membrane proteins A (OmpA) and X (OmpX) are essential for basolateral invasion of *Cronobacter sakazakii*. *Appl. Environ. Microbiol.* 76 5188–5198. 10.1128/AEM.02498-09 20543055 PMC2916488

[B23] Kucerova E. Clifton S. W. Xia X. Q. Long F. Porwollik S. Fulton L. (2010). Genome sequence of *Cronobacter sakazakii* BAA-894 and comparative genomic hybridization analysis with other Cronobacter species. *PLoS One* 5:e9556. 10.1371/journal.pone.0009556 20221447 PMC2833190

[B24] Lee H. Hsu F.-F. Turk J. Groisman E. A. (2004). The PmrA-regulated pmrC gene mediates phosphoethanolamine modification of lipid A and polymyxin resistance in *Salmonella enterica*. *J. Bacteriol.* 186 4124–4133. 10.1128/JB.186.13.4124-4133.2004 15205413 PMC421605

[B25] Lehner A. Riedel K. Eberl L. Breeuwer P. Diep B. Stephan R. (2005). Biofilm formation, extracellular polysaccharide production, cell-to-cell signaling, in various *C.sakazakii* strains: aspects promoting environmental persistenc. *Food Protoc.* 68:7 10.1002/9783527629237.ch1 16300064

[B26] Li Y. Yoon S. H. Wang X. Ernst R. K. Goodlett D. R. (2016). Structural derivation of lipid A from Cronobacter sakazakii using tandem mass spectrometry. *Rapid Commun. Mass Spectr.* 30 2265–2270. 10.1002/rcm.7712 27502448

[B27] Liu L. Li Y. Wang X. Guo W. (2016). A phosphoethanolamine transferase specific for the 4’-phosphate residue of *Cronobacter sakazakii* lipid A. *J. Appl. Microbiol.* 121 1444–1456. 10.1111/jam.13280 27564119

[B28] Livak K. J. Schmittgen T. D. (2001). Analysis of relative gene expression data using real-time quantitative PCR and the 2-ΔΔCT method. *Methods* 25 402–408. 10.1006/meth.2001.1262 11846609

[B29] McPhee J. B. Bains M. Winsor G. Lewenza S. Kwasnicka A. Brazas M. D. (2006). Contribution of the PhoP-PhoQ and PmrA-PmrB two-component regulatory systems to Mg2+-induced gene regulation in *Pseudomonas aeruginosa*. *J. Bacteriol.* 188 3995–4006. 10.1128/JB.00053-06 16707691 PMC1482896

[B30] Mitrophanov A. Y. Jewett M. W. Hadley T. J. Groisman E. A. (2008). Evolution and dynamics of regulatory architectures controlling polymyxin B resistance in enteric bacteria. *PLoS Genet.* 4:e1000233. 10.1371/journal.pgen.1000233 18949034 PMC2565834

[B31] Murray S. R. Ernst R. K. Bermudes D. Miller S. I. Low K. B. (2007). pmrA(Con) confers pmrHFIJKL-dependent EGTA and polymyxin resistance on msbB *Salmonella* by decorating lipid A with phosphoethanolamine. *J. Bacteriol.* 189 5161–5169. 10.1128/JB.01969-06 17449614 PMC1951887

[B32] Raetz C. R. Whitfield C. (2002). Lipopolysaccharide endotoxins. *Annu. Rev. Biochem.* 71 635–700. 10.1146/annurev.biochem.71.110601.135414 12045108 PMC2569852

[B33] Roland K. L. Martin L. E. Esther C. R. Spitznagel J. K. (1993). Spontaneous pmrA mutants of *Salmonella* typhimurium LT2 define a new two-component regulatory system with a possible role in virulence. *J. Bacteriol.* 175 4154–4164. 10.1128/jb.175.13.4154-4164.1993 8391535 PMC204845

[B34] Townsend S. Hurrell E. Forsythe S. (2008). Virulence studies of *Enterobacter sakazakii* isolates associated with a neonatal intensive care unit outbreak. *BMC Microbiol.* 8:64. 10.1186/1471-2180-8-64 18423002 PMC2386127

[B35] Townsend S. M. Hurrell E. Gonzalez-Gomez I. Lowe J. Frye J. G. Forsythe S. (2007). *Enterobacter sakazakii* invades brain capillary endothelial cells, persists in human macrophages influencing cytokine secretion and induces severe brain pathology in the neonatal rat. *Microbiology* 153 3538–3547. 10.1099/mic.0.2007/009316-0 17906151

[B36] Viau C. Le Sage V. Ting D. K. Gross J. Le Moual H. (2011). Absence of PmrAB-mediated phosphoethanolamine modifications of *Citrobacter rodentium* lipopolysaccharide affects outer membrane integrity. *J. Bacteriol.* 193 2168–2176. 10.1128/JB.01449-10 21378194 PMC3133075

[B37] Wang B. Han Y. Li Y. Li Y. Wang X. (2015). Immuno-stimulatory activity of *Escherichia coli* mutants producing Kdo2-monophosphoryl-lipid A or Kdo2-pentaacyl-monophosphoryl-lipid A. *PLoS One* 10:e0144714. 10.1371/journal.pone.0144714 26710252 PMC4692390

[B38] Wang X. Quinn P. J. Yan A. (2015). Kdo2-lipid A: structural diversity and impact on immunopharmacology. *Biol. Rev. Camb. Philos. Soc.* 90 408–427. 10.1111/brv.12114 24838025 PMC4402001

[B39] Wang Z. Wang J. Ren G. Li Y. Wang X. (2015). Influence of core oligosaccharide of lipopolysaccharide to outer membrane behavior of *Escherichia coli*. *Mar. Drugs* 13 3325–3339. 10.3390/md13063325 26023839 PMC4483631

[B40] Wang J. Ma W. Wang Z. Li Y. Wang X. (2014). Construction and characterization of an *Escherichia coli* mutant producing Kdo2-lipid A. *Mar. Drugs* 12 1495–1511. 10.3390/md12031495 24633251 PMC3967223

[B41] Wang L. Wang Q. Reeves P. R. (2010). *Endotoxins: Structure, Function and Recognition.* Berlin: Springer.

[B42] Winfield M. D. Groisman E. A. (2004). Phenotypic differences between *Salmonella* and *Escherichia coli* resulting from the disparate regulation of homologous genes. *Proc. Natl. Acad. Sci. U.S.A.* 101 17162–17167. 10.1073/pnas.0406038101 15569938 PMC534605

[B43] Winfield M. D. Latifi T. Groisman E. A. (2005). Transcriptional regulation of the 4-amino-4-deoxy-L-arabinose biosynthetic genes in *Yersinia pestis*. *J. Biol. Chem.* 280 14765–14772. 10.1074/jbc.M413900200 15710615

[B44] Wosten M. Groisman E. (1999). Molecular characterization of the PmrA regulon. *J. Biol. Chem.* 274:38. 10.1074/jbc.274.38.27185 10480935

[B45] Wosten M. Kox L. Chamnongpol S. Soncini F. Groisman E. (2000). A signal transduction system that responds to extracellular iron. *Cell* 103 113–125. 10.1016/s0092-8674(00)00092-1 11051552

[B46] Zavaglia G. A. Kociubinski G. Pérez P. Disalvo E. De Antoni G. (2002). Effect of bile on the lipid composition and surface properties of bifidobacteria. *J. Appl. Microbiol.* 93 794–799. 10.1046/j.1365-2672.2002.01747.x 12392525

[B47] Zhang H. Li Y. Wang C. Wang X. (2018). Understanding the high L-valine production in *Corynebacterium glutamicum* VWB-1 using transcriptomics and proteomics. *Sci. Rep.* 8:3632. 10.1038/s41598-018-21926-5 29483542 PMC5827029

[B48] Zhou Z. Ribeiro A. A. Lin S. Cotter R. J. Miller S. I. Raetz C. R. (2001). Lipid A modifications in polymyxin-resistant *Salmonella* typhimurium: PMRA-dependent 4-amino-4-deoxy-L-arabinose, and phosphoethanolamine incorporation. *J. Biol. Chem.* 276 43111–43121. 10.1074/jbc.M106960200 11535603

